# Supplementary material for: Disrupted in renal carcinoma 2 (DIRC2/SLC49A4) is an H+-driven lysosomal pyridoxine exporter
Source: Life Sci Alliance. 2022 Dec 1;6(2):e202201629. doi: 10.26508/lsa.202201629 (PMC9719028; doi:10.26508/lsa.202201629)
Supplement: Supplementary file 1 [file LSA-2022-01629_TableS1.docx]

**Supplementary Table 1** Primers for amplification of the cDNA for DIRC2

Solid line and broken line are restriction sites for *EcoRI* and *XbaI*, respectively.

| PCR | Orientation | Sequence (5′–3′) |
| --- | --- | --- |
| 1st | Forward | ACTATTCTGCGCTGGGCTAGTC |
|  | Reverse | TTCACCACCTTGGCTGATAAGA |
| 2nd | Forward | GCAGAATTCCGCCATGGGCTCTCGCTG |
|  | Reverse | GCTTCTAGATTAAACGGAGACAACCA |
