# Supplementary material for: Disrupted in renal carcinoma 2 (DIRC2/SLC49A4) is an H+-driven lysosomal pyridoxine exporter
Source: Life Sci Alliance. 2022 Dec 1;6(2):e202201629. doi: 10.26508/lsa.202201629 (PMC9719028; doi:10.26508/lsa.202201629)
Supplement: Supplementary file 2 [file LSA-2022-01629_TableS2.docx]

**Supplementary Table 2**  Primers for generation of the cDNA for DIRC2-AA

| Orientation | Sequence (5′–3′) |
| --- | --- |
| Forward | ACTATTCTGCGCTGGGCTAGTC |
| Reverse | TTCACCACCTTGGCTGATAAGA |
