# Supplementary material for: Disrupted in renal carcinoma 2 (DIRC2/SLC49A4) is an H+-driven lysosomal pyridoxine exporter
Source: Life Sci Alliance. 2022 Dec 1;6(2):e202201629. doi: 10.26508/lsa.202201629 (PMC9719028; doi:10.26508/lsa.202201629)
Supplement: Supplementary file 3 [file LSA-2022-01629_TableS3.docx]

**Supplementary Table 3**  Sequences of the siRNAs for DIRC2

| Type | Sequence (5′–3′) |
| --- | --- |
| Sense | GAUGCAUACCUAUAUCAGAtt |
| Antisense | UCUGAUAUAGGUAUGCAUCtt |
