# Supplementary material for: Disrupted in renal carcinoma 2 (DIRC2/SLC49A4) is an H+-driven lysosomal pyridoxine exporter
Source: Life Sci Alliance. 2022 Dec 1;6(2):e202201629. doi: 10.26508/lsa.202201629 (PMC9719028; doi:10.26508/lsa.202201629)
Supplement: Supplementary file 4 [file LSA-2022-01629_TableS4.docx]

**Supplementary Table 4**  Primers for real-time PCR analysis of mRNA expression

| Protein | Orientation | Sequence (5′–3′) |
| --- | --- | --- |
| DIRC2 | Forward | TTATTTCCCACCCCGACCTCC |
|  | Reverse | AGACCAGCCAGCAAATACACC |
| THTR1 | Forward | CAAGTTACTGTCGAAGTGCCA |
|  | Reverse | GCAGGTAGAAGGAATGTGGTGAA |
| THTR2 | Forward | TCCCCGTTTGGACATACTCCT |
|  | Reverse | GCTTGTAGCGGACATAATCGGT |
| UBC | Forward | CTGGAAGATGGTCGTACCCTG |
|  | Reverse | GGTCTTGCCAGTGAGTGTCT |
| GAPDH | Forward | CGGAGTCAACGGATTTGGTCGTAT |
|  | Reverse | AGCCTTCTCCATGGTGGTGAAGAC |
